# The efficacy and safety of acupuncture for perimenopausal insomnia: A protocol for a network meta-analysis

**DOI:** 10.1097/MD.0000000000023741

**Published:** 2020-12-24

**Authors:** Zhao Wang, Fengting Zhai, Xiaomin Zhao, Guizhi Zhao, Na Li, Fang Zhang, Jinxing Liu

**Affiliations:** a First College of Clinical Medicine, Shandong University of Traditional Chinese Medicine; b Shandong college of Traditional Chinese Medicine; c The Affiliated Hospital of Shandong University of Traditional Chinese Medicine.

**Keywords:** acupuncture, network meta-analysis, perimenopausal insomnia, protocol

## Abstract

**Background:**

As a common symptom of perimenopausal period, perimenopausal insomnia brings great pain to many women and families. Acupuncture has been accepted by people as the incidence rate of this disease increases. The purpose of this study is to systematically compare the safety and efficacy of various acupuncture treatments for perimenopausal insomnia through network meta-analysis.

**Methods:**

We will search Web of Science, PubMed, The Cochrane Library, Embase, Chinese National Knowledge Infrastructure (CNKI), Wan Fang Date, VIP database, conference papers and grey literature. All relevant Randomized controlled trial (RCT) using acupuncture for perimenopausal insomnia will be included. Two reviewers will independently search and screen date. Network meta-analysis will be completed by Stata and WinBUGS software.

**Results:**

This study will compare the efficacy and safety of different acupuncture treatments for perimenopausal insomnia.

**Conclusion:**

The result of this study will provide reliable evidence for evaluating the efficacy and safety of acupuncture in the treatment of perimenpausal insomnia.

**INPLASY registration number:**

INPLASY2020110047.

## 1 Introduction

As we know, menopause is a vexing problem for most people, because it always comes with hot flash, sweating, depression, insomnia, osteoporosis and so on.^[1]^ Of all the above symptoms, perimenopausal insomnia has become the most concerned problem.^[2]^ It is mainly because it has a negative impact on patients daytime function^[2]^, reduces the quality of life of patients^[1–4]^ and causes economic losses^[4–6]^. According to the National Institutes of Health statement, the prevalence of sleep disturbance varies from 39% to 47% in perimenopause,^[7]^ and the proportion increases significantly with age,^[5,7–10]^ which remains elevated in postmenopause.^[7,10]^

Perimenopausal insomnia is characterized by having trouble falling asleep, early awakening and difficulty in falling asleep after waking.^[11–12]^ At present, although the mechanism of perimenopausal insomnia is not completely clear, it is generally attribute to hypothalamic-pituitary-ovarian axis disorder, which results in vegetative nerve functional disturbance. In addition, unique symptoms of perimenopause, such as hot flashes, night sweats, bone aches, anxiety and depression also contribute to sleep disturbance.^[2,5,7]^ At the specific time of perimenopause, most women act as parent, wife and child simultaneously, they need to take care of their aging parents, immature children, and shoulder most of the housework.^[12,13]^ At the same time, these women who are climbing the career ladder^[14]^ need to put more energy into work challenge. It is not difficult to understand, the pressure from family and society is a significant factor to perimenopausal insomnia.^[7,13]^

For perimenopausal insomnia, the main treatment methods are hormone replacement therapy (HRT) and sedative and hypnotic drugs. HRT is the first-line therapy for perimenopausal syndrome.^[5]^ Estrogen is known to exert effects on sleep-related factors,^[15]^ therefore, estrogen can also be used alone or in combination with progesterone.^[16]^ Sedative-hypnotic drugs include benzodiazepines^[17]^ and non-benzodiazepine hypnotics^[16]^ (e.g., zaleplon, zolpidem, zopiclone, eszopiclone).^[18]^ Although these drugs are a great boon for perimenopausal insomnia sufferers, in the long run, patients will become dependent on drug^[17,19]^ and can increase the risk of endometrial cancer, breast cancer, stroke, and coronary heart diseases.^[19,20]^ So, more and more doctors and patients are looking for a safer and more effective therapy.^[17,21]^

As one of therapeutic methods of Traditional Chinese Medicine, acupuncture is well accepted by world people^[10,21–23]^ and widely used in various diseases.^[17,18]^ A number of randomized controlled trials and meta-analysis have shown acupuncture can improve peoples sleep quality in perimenopause.^[11,24]^ Studies have found that the increase of serum estradiol levels is a possible mechanism of acupuncture in alleviating perimenopausal insomnia.^[24]^ Positive regulation of the sleep architecture and mood disorder might be able to improve sleep disturbances, after the sleep-related center responded acupuncture signal.^[17]^ However, the exact physiological or biochemical mechanism of acupuncture improving sleep is still not completely understood.^[18]^

Acupuncture has different forms, including body acupuncture, electroacupuncture, warm acupuncture, ear acupuncture, abdominal acupuncture, moxibustion cupping, massage, acupoint embedding thread, acupoint application and so on.^[18]^ Doctors choose different kinds of acupuncture according to different conditions of patients.^[25]^

## 2 Objectives

The purpose of this study is to provide a safer and more effective treatment for perimenopausal insomnia by comparing the clinical efficacy of different acupuncture.

## 3 Materials and methods

With the guidance of Preferred Reporting Items for Systematic Review and Meta-Analysis Protocols guidelines we has completed our network meta-analysis (NMA) protocol, and has been registered in the International Platform of Registered Systematic Review and Meta-analysis Protocols (INPLASY) with the number is INPLASY2020110047.

### 3.1 Selection criteria

#### 3.1.1 Types of studies

All relevant Randomized controlled trial (RCT) using acupuncture for perimenopausal insomnia will be included.

#### 3.1.2 Types of patients

Women who meet insomnia in perimenopause, and are clearly diagnosed as peimenopausal insomnia will be included without restrictions on race, nationality, education and economic incomes.

#### 3.1.3 Intervention and comparators

All randomized controlled trials with acupuncture therapy (without restriction on form, acupoint selection, operation method, etc.) will be included. The control group with other treatment (sham acupuncture, placebo, drugs, etc.) will be included.

#### 3.1.4 Outcomes

Main outcome is sleep quality measured by the Pittsburgh Sleep Quality Index Scale (PSQI). Additional outcomes include quality of life measured by Quality of Life Scale (SF-36), hormone levels and adverse events.

### 3.2 Database search strategy

We will search Web of Science, PubMed, The Cochrane Library, Embase, Chinese National Knowledge Infrastructure (CNKI), Wan Fang Date and VIP database. Meanwhile, conference papers and grey literature will be searched to get RCTs of acupuncture for perimenopausal insomnia. Relevant studies published before October 2020 will be included. The search terms will combine Medical Subject Heading (MeSH) with free-text terms, including perimenopausal insomnia, perimenopausal sleep disorder, perimenopausal sleep disturbance, acupuncture, electroacupuncture, auriculotherapy, fire needling, warm acupuncture, moxibustion, etc. The search strategy of PubMed is shown in Table 1.

**Table 1:** Search strategy used in PubMed database.

No. Search item
#1 Perimenopaus^∗^[Mesh]
#2 Menopause [Title/Abstract] OR Climacteric[Title/Abstract]
#3 #1 OR #2
#4 Insomnia [Mesh]
#5 Sleep disorder [Title/Abstract] OR Sleep disturbance [Title/Abstract] OR
Sleeplessness [Title/Abstract]
#6 #4 OR #5
#7 Acupuncture [Mesh]
#8 Acupuncture therapy [Title/Abstract] OR Electroacupuncture [Title/Abstract] OR Acupuncture points [Title/Abstract] OR Auriculotherapy [Title/Abstract] OR Fire needling [Title/Abstract] OR Warm acupuncture [Title/Abstract] OR Moxibustion [Title/Abstract]
#9 #7 OR #8
#10 Randomized controlled trial [Publication Type] OR Controlled clinical trial [Publication Type]
#11 Randomly [Title/Abstract] OR Randomized [Title/Abstract] Randomly allocation [Title/Abstract]
#12 #10 OR #11
#13 #3 AND #6 AND #9 AND #12

### 3.3 Study selection and date extraction

According to a prepared date extraction form, 2 reviewers (Zhao Wang and Fengting Zhai) will independently search and screen date including studies characteristics (title, authors, journal, publication time, method of randomization and blinding), participants characteristics (age, duration of disease, diagnostic criteria and sample size), interventions and controls, outcomes. Any disagreement will be resolved through discussion with a third reviewer (Xiaomin Zhao). If the information is incomplete, we will contact author by email or telephone. Reviewers eliminate literature by Note Express, exclude irrelevant literature through reading the title and abstract, and include the required literature after reading the full text.

### 3.4 Risk of bias assessment

Two reviewers (Zhao Wang and Fengting Zhai) will independently assess the risk of bias of included studies according to The Cochrane Handbook for systematic Review of Interventions. Seven domains will be included, random sequence generation, allocation concealment, blinding of participants and personnel, blinding of outcome assessment, incomplete outcome date, selective outcome reporting and other bias. Each item will be judged as high, low and unclear (Xiaomin Zhao).

### 3.5 Statistical analysis

Revman5.3 will be used to evaluate the bias risk. Heterogeneity test depends on *P* and *I*^2^. If *P* ≥ .05 and *I*^2^ ≤ 50%, it means there is no obvious heterogeneity of included studies, then fixed-effected model would be adopted. If *P* < .05 and *I*^2^ > 50%, it indicates there is heterogeneity, then random effect model would be selected and a subgroup analysis would be conducted to analyze the source of heterogeneity. If the heterogeneity is large and the source is unknown, only descriptive analysis will be performed. Mean different (MD) will be used for the continuous date, the odds rations (OR) will be used for dichotomous date. Both continuous and dichotomous dates will be present 95% confidence intervals (CL).

Stata 15.0 and WinBUGS 1.4.3 software will be used to perform this network meta-analysis, using different commands to realize network evidence plot construction and ranking between direct comparison interventions and indirect comparison interventions. The node-splitting method and loop inconsistency will be used to test inconsistency of the network. The surface under the cumulative ranking area (SUCRA) will be used to calculate the probability ranking of the effects of various treatments. The higher value of SUCRA, the better effect of the intervention.

### 3.6 Sensitivity analysis

We will conduct sensitivity analysis of main outcomes to ensure reliability of conclusion, including risk of bias, missing state and methodological quality.

### 3.7 Assessment of publication bias and evidence quality

The funnel plot will be used to evaluate publication bias. If the funnel plot is symmetric, there is no publication bias, otherwise, it suggests existing publication bias.

The Grading of Recommendations Assessment, Development and Evaluation (GRADE) will be used to assess the quality of evidence, including risk of bias, indirectness, inconsistency, imprecision and publication bias.

## 4 Discussion

As a traditional Chinese treatment, acupuncture is constantly evolving and changing.^[26]^ Researchers have found that acupuncture has some advantages over drugs in treating perimenopausal insomnia. However, there are many kinds of acupuncture, which one is the best? Numerous meta-analysis studies focused on the comparison of drug application and acupuncture treatment,^[27]^ or between 2 kinds of acupuncture,^[28]^ but rarely comparisons with different kinds of acupuncture. This study will complement this deficiency through network meta-analysis, evaluate the efficacy and safety of acupuncture in the treatment of perimenopausal insomnia and rank the curative effect of all kinds of acupuncture to provide the most effective treatment for the clinical application.

However, this study also has some limitations. For example, due to language barrier, only English and Chinese documents can be searched. In addition, different kinds of acupuncture and different acupoint selection^[28]^ may cause significant heterogeneity. Therefore, it is necessary to carry out a higher quality research in the future.

## Author contributions

**Conceptualization:** Zhao Wang, Jinxing Liu.

**Data curation:** Zhao Wang, Fengting Zhai, Jinxing Liu.

**Formal analysis:** Zhao Wang, Fengting Zhai, Xiaomin Zhao.

**Funding acquisition:** Jinxing Liu.

**Investigation:** Zhao Wang.

**Methodology:** Zhao Wang, Fengting Zhai, Xiaomin Zhao.

**Project administration:** Zhao Wang.

**Resources:** Guizhi Zhao, Na Li, Fang Zhang.

**Software:** Zhao Wang, Fengting Zhai, Guizhi Zhao, Fang Zhang.

**Supervision:** Zhao Wang.

**Validation:** Zhao Wang.

**Visualization:** Zhao Wang.

**Writing – original draft:** Zhao Wang, Fengting Zhai, Li Na.

**Writing – review & editing:** Zhao Wang, Jinxing Liu.

## References

[1] Li Y Zheng H Zheng Q Use acupuncture to relieve perimenopausal syndrome: study protocol of a randomized controlled trial. Trials 2014;15:198–207. 24886348 10.1186/1745-6215-15-198 PMC4055374

[2] Caruso D Masci I Cipollone G Insomnia and depressive symptoms during the menopausal transition: theoretical and therapeutic implications of a self-reinforcing feedback loop. Maturitas 2019;123:78–81. 31027682 10.1016/j.maturitas.2019.02.007

[3] Tal JZ Suh SA Dowdle CL Treatment of insomnia, insomnia symptoms, and obstructive sleep apnea during and after menopause: therapeutic approaches. Curr Psychiatry Rev 2015;11:63–83. 26478725 10.2174/1573400510666140929194848 PMC4607064

[4] Bolge SC Balkrishnan R Kannan H Burden associated with chronic sleep maintenance insomnia characterized by nighttime awakenings among women with menopausal symptoms. Menopause 2010;17:80–6. 19730276 10.1097/gme.0b013e3181b4c286

[5] Polo-Kantola P Saaresranta T Polo O Aetiology and treatment of sleep disturbances during perimenopause and postmenopause. Cns Drugs 2001;15:445–52. 11524023 10.2165/00023210-200115060-00003

[6] Fu C Zhao N Liu Z Acupuncture improves peri-menopausal insomnia: a randomized controlled trial. Sleep 2017;40 doi: 10.1093/sleep/zsx153. 10.1093/sleep/zsx153 29029258

[7] Kravitz HM Joffe H Sleep during the perimenopause: a SWAN story. Obstet Gyn Clin N Am 2011;38:567–86. 10.1016/j.ogc.2011.06.002 PMC3185248 21961720

[8] National Institutes of Health State-of-the-Science Conference Statement: Management of Menopause-Related Symptoms. J Urol 2006;175:659–60. 15968015

[9] van Dijk GM Kavousi M Troup J Health issues for menopausal women: the top 11 conditions have common solutions. Maturitas 2015;80:24–30. 25449663 10.1016/j.maturitas.2014.09.013

[10] Hachul H Garcia TK Maciel AL Acupuncture improves sleep in postmenopause in a randomized, double-blind, placebo-controlled study. Climacteric 2013;16:36–40. 22943846 10.3109/13697137.2012.698432

[11] Clinical challenges of perimenopause: consensus opinion of The North American Menopause Society. Menopause 2000;7:5–13. 10646698

[12] Ensrud KE Joffe H Guthrie KA Effect of escitalopram on insomnia symptoms and subjective sleep quality in healthy perimenopausal and postmenopausal women with hot flashes: a randomized controlled trial. Menopause 2012;19:848–55. 22433978 10.1097/gme.0b013e3182476099 PMC3382013

[13] Triebner K Johannessen A Puggini L Menopause as a predictor of new-onset asthma: a longitudinal Northern European population study. J Allergy Clin Immun 2016;137:50–7. 26435006 10.1016/j.jaci.2015.08.019

[14] Santoro N Perimenopause: from research to practice. J Women's Health 2016;25:332–9. 10.1089/jwh.2015.5556 PMC4834516 26653408

[15] Soares CN Joffe H Rubens R Eszopiclone in patients with insomnia during perimenopause and early postmenopause: a randomized controlled trial. Obstet Gynecol 2006;108:1402–10. 17138773 10.1097/01.AOG.0000245449.97365.97

[16] Avis NE Coeytaux RR Isom S Acupuncture in Menopause (AIM) study: a pragmatic, randomized controlled trial. Menopause 2016;23:626–37. 27023860 10.1097/GME.0000000000000597 PMC4874921

[17] Wu X Zhang W Qin Y Effect of acupuncture and its influence on cerebral activity in perimenopausal insomniacs: study protocol for a randomized controlled trial. Trials 2017;18:377–86. 28806907 10.1186/s13063-017-2072-7 PMC5557519

[18] Cheuk DK Yeung WF Chung KF Acupuncture for insomnia. Cochrane Database Syst Rev 2012;12:CD005472. 10.1002/14651858.CD005472.pub2 17636800

[19] National Institutes of Health. National Institutes of Health State of the Science Conference statement on Manifestations and Management of Chronic Insomnia in Adults, June 13–15, 2005. Sleep 2005;28:1049–57. 16268373 10.1093/sleep/28.9.1049

[20] Marjoribanks J Farquhar C Roberts H Long-term hormone therapy for perimenopausal and postmenopausal women. Cochrane Database Syst Rev 2017;1:CD004143. 28093732 10.1002/14651858.CD004143.pub5 PMC6465148

[21] Baumelou A Liu B Wang XY Perspectives in clinical research of acupuncture on menopausal symptoms. Chin J Integr Med 2011;17:893–7. 22139540 10.1007/s11655-011-0930-9

[22] Burke A Upchurch DM Dye C Acupuncture use in the United States: findings from the National Health Interview Survey. J Altern Complement Med 2006;12:639–48. 16970534 10.1089/acm.2006.12.639

[23] Kaptchuk TJ Acupuncture: theory, efficacy, and practice. Ann Intern Med 2002;136:374. 11874310 10.7326/0003-4819-136-5-200203050-00010

[24] Chiu HY Hsieh YJ Tsai PS Acupuncture to reduce sleep disturbances in perimenopausal and postmenopausal women: a systematic review and meta-analysis. Obstet Gynecol 2016;127:507–15. 26855097 10.1097/AOG.0000000000001268

[25] Zhao J Interpretation of acupuncture theory from acupuncture application. Zhongguo Zhen Jiu 2017;37:1115–8. 29354983 10.13703/j.0255-2930.2017.10.024

[26] Zhuang Y Xing JJ Li J History of acupuncture research. Int Rev Neurobiol 2013;111:1–23. 24215915 10.1016/B978-0-12-411545-3.00001-8

[27] Meng F Duan PB Zhu J Effect of Gua sha therapy on perimenopausal syndrome: a randomized controlled trial. Menopause 2017;24:299–307. 27760084 10.1097/GME.0000000000000752

[28] Zhang WR Guo H Tan SH Moxibustion therapy is superior to manual acupuncture in the treatment of perimenopausal insomnia: a randomized controlled trial. Zhen Ci Yan Jiu 2019;44:358–62. 31155869 10.13702/j.1000-0607.180320

